# Balancing the equation: assessing the impact of management practices on staff and faculty wellbeing in Chinese higher education institutions

**DOI:** 10.3389/fpsyg.2024.1385612

**Published:** 2024-05-31

**Authors:** Tingting Hu

**Affiliations:** School of Chemical Engineering, Huaiyin Institute of Technology, Jiangsu, China

**Keywords:** human resource management practices, Chinese higher education institutions, faculty, administrative staff, job satisfaction, organizational justice, work-family culture

## Abstract

**Introduction:**

The intersection of work-family dynamics and job contentment has become a pivotal area of investigation within the higher education landscape, drawing scholarly attention, especially in the Chinese context. This study delves into the intricate relationship between work-family culture and job satisfaction, with a particular spotlight on the mediating influence of perceptions of organizational fairness. The impetus behind this emphasis lies in the burgeoning acknowledgment of organizational justice as a pivotal force shaping employee attitudes and conduct within academic establishments.

**Method:**

This research was conducted using two distinct groups. The first group consisted of 1,075 faculty members at Chinese universities, while the second group comprised 972 administrative and technical employees at these institutions.

**Results:**

The mediational analyses provided in this study offer an enhanced comprehension of the intricate relationships under discussion. Significantly, the findings reveal that Work-Family Culture plays a crucial predictive role in influencing perceptions of Organizational Justice among both faculty and administrative staff. More importantly, the study uncovers that Work-Family Culture indirectly affects Job Satisfaction through its impact on Organizational Justice.

**Discussion:**

This insight underscores the importance of harmonious work-family interactions as a determinant of job-related attitudes and satisfaction levels.

## Introduction

In the realm of higher education, the interplay between work-family culture and job satisfaction, particularly among faculty and administrative staff, has emerged as a focal point of empirical inquiry, specifically in China (Shang et al., 2021). This paper aims to investigate into the relationship between these two constructs, with a specific emphasis on the mediating role of organizational justice perceptions. The rationale for this focus stems from the growing recognition of organizational justice as a critical determinant of employee attitudes and behaviors within academic institutions (Shen et al., 2022).

Work-family culture, characterized by organizational norms and practices that support the integration of employees’ work and family lives, has been extensively studied for its impact on job satisfaction. However, the mechanisms through which this impact is exerted remain underexplored (Shahid et al., 2022). Organizational justice, which encompasses perceptions of fairness in organizational procedures, interactions, and outcomes, is posited to be a key mediating factor in this relationship. It addresses how equitably and respectfully individuals are treated within their work environment and how these perceptions influence their overall job satisfaction (Zhang, 2023).

Empirical studies have shed light on various aspects of this relationship. Shahid et al. (2022) underscored the significance of perceived organizational support in enhancing job satisfaction, linking it to high-performance work systems in university settings. Similarly, the seminal work of Judge and Colquitt (2004) highlighted the strong association between procedural and interpersonal justice and reduced stress levels, mediated by work–family conflict. These findings suggest a complex interplay between organizational justice, work-family culture, and job satisfaction, warranting a comprehensive examination (Zhu et al., 2016).

Therefore, this paper seeks to synthesize these strands of research to offer a cohesive understanding of how organizational justice perceptions act as a conduit through which work-family culture affects job satisfaction among university faculty and administrative staff. Through this exploration, we aim to contribute to the broader discourse on employee wellbeing in academic environments and provide actionable insights for university administrations.

## Literature review

### Human resource management practices in Chinese higher education institutions

Human Resource Management (HRM) practices in higher education institutions have increasingly come under scrutiny, especially in the context of Chinese universities (Lin and Xu, 2020). This scrutiny is primarily due to the changing nature of academic work and the recent challenges faced by staff in these institutions. The role of HRM in influencing the wellbeing of employees in Chinese higher education has been highlighted in several studies, reflecting a growing awareness of its importance.

Zeng and Chen (2020) emphasize the transformation in Chinese higher education institutions, particularly in response to global educational trends and local workforce needs. This transformation has necessitated a reevaluation of HRM practices to ensure they are aligned with the changing demands of academic and administrative staff. The effectiveness of HRM practices in enhancing job satisfaction and overall wellbeing among university staff is a recurring theme in their analysis (Kloutsiniotis and Mihail, 2018).

Furthermore, Yu et al. (2020) explore the specific HRM strategies employed by Chinese universities. A range of practices, including talent acquisition, development, and retention strategies are tailored to the unique context of higher education. This line of research points to the positive impact of these HRM practices on employee engagement and job satisfaction, which are crucial for the wellbeing of both academic and administrative staff.

The relevance of HRM practices for academic staff is particularly significant, as highlighted by Williams (2020). The academic staff in Chinese higher education institutions face unique pressures, including research productivity, teaching excellence, and administrative responsibilities. Effective HRM practices can help mitigate these pressures by providing support, development opportunities, and a conducive work environment (Saleh et al., 2023). This, in turn, can enhance the overall wellbeing and job satisfaction of academic staff (Zakariya and Wardat, 2023), a topic that deserves current attention due to its impact on students’ achievement and Higher Education Institutions overall performance (Hidayat and Wardat, 2023).

For administrative staff, HRM practices play a similarly vital role, as discussed by Chung et al. (2010). The administrative staff in Chinese universities often grapple with role ambiguity and workload challenges. HRM practices that focus on clarity of roles, skill development, and workload management can significantly improve their work experience and wellbeing.

To conclude, considering the significance of both professional categories in ensuring the effective operation of Higher Education Institutions, and the imperative of safeguarding employee wellbeing, investigating HRM practices and their influence on job satisfaction and work capability is pivotal. This research enhances comprehension of this multifaceted domain and guides interventions grounded in theoretical knowledge.

### Work-family culture from a Confucian perspective

Despite that Westernization and modernization have significantly influenced China’s current culture, Confucian principles remain its bedrock (Warner, 2010). The essence of Confucianism was based through seven key values (Redding, 2002): Societal Order (Upholding established societal structures and fostering harmony), Hierarchy (Acceptance of a paternalistic approach in families and organizations), Reciprocity (Focusing on trust within social exchanges), Control (Distribution of resources based on the discretion of superiors), Insecurity (The necessity to safeguard against adverse conditions and to accumulate reserves), Family-based Collectivism (Emphasizing care for the extended family, valuing filial piety and family reputation), and Knowledge (Placing a high value on learning, intelligence, education, and resourcefulness). These values deeply infiltrate all aspects of Chinese society, influencing individual perspectives on work and family, and consequently shaping the work-life experiences of Chinese workers. To sum up, this set of values modulate the perceptions of HRM practices about the interface among work and family.

Thompson et al. (1999) defined a supportive work-family culture as “the shared assumptions, beliefs, and values regarding the extent to which an organization supports and values the integration of employees’ work and family lives.” Later studies emphasize that such a culture offers flexibility, tolerance, and support for family obligations, leading to increased productivity and personal wellbeing (Fiksenbaum, 2014). Furthermore, Thompson et al. (1999) suggest that work-family programs are effective only within an organizational culture that supports them. Research in this area has consistently indicated that supportive work-family cultures decrease employees’ perception of work–family conflict and mitigate potential negative outcomes. Fiksenbaum (2014) highlights that such cultures are a source of employee satisfaction and wellbeing both at work and home. Premeaux et al. (2007) connect supportive work-family culture to the theory of Conservation of Resources (COR), arguing that it increases individuals’ pool of resources, leading to reduced stress and conflict regarding work-family intervention.

Recent studies also contribute significantly to this discussion from a Confucian perspective. Chang et al. (2024) demonstrate how Confucian work values can moderate the relationship between workplace culture and innovative behavior, highlighting the importance of perspective-taking and knowledge-sharing in the context of companionate love culture. Additionally, Zhang (2023) illustrates the integration of Confucian culture in the education of college students, promoting the development of “junzi” or gentlemen, which reflects a deep-rooted Confucian influence on work-family dynamics in educational settings. Pan and Shang (2023) explore how Confucian culture affects family travel behavior, indicating its impact on parental concern and psychological wellbeing. Liu et al. (2022) discuss how Confucian values like face consciousness and group conformity influence family-related economic decisions, such as housing consumption, which are integral to understanding the work-family interface In summary, the literature suggests that a supportive work-family culture, influenced by both organizational and cultural factors, plays a crucial role in enhancing employee wellbeing and balancing work and family responsibilities. The integration of Confucian principles into this discussion provides a richer understanding of the cultural nuances that influence work-family dynamics.

### Job satisfaction of faculty and administrative staff at universities

Despite that Job satisfaction is one the most researched constructs, some subtle process that could impact it, and specifically differentiate the perceptions among faculty and administrative staff, can be taken into account.

High-performance work systems are found to have a positive impact on job satisfaction among university faculty and staff. Shahid et al. (2022) highlighted the importance of perceived organizational support in enhancing the effectiveness of these systems. This suggests that when employees feel supported by their organization, they are more likely to experience job satisfaction, irrespective of their role within the university.

The relationship between human resource management practices and job performance is another key area of focus also in non-Western societies. Recent studies found that satisfaction with HRMPs at Kyambogo University was moderately related to the job performance of academic staff, implying that effective HRMPs can positively influence both satisfaction and performance (Kasule et al., 2022).

Organizational culture, particularly in the context of digital transformation, plays a crucial role in shaping job satisfaction. Shoaib (2022) discovered a positive correlation between organizational culture and job satisfaction, with digital transformation serving as a mediating variable. This indicates that embracing digital advancements in organizational practices can enhance job satisfaction among university staff.

Job security, especially among non-teaching staff, is another significant factor. Recent research emphasized the crucial role of job security in influencing job satisfaction for non-teaching staff at public universities in Lagos State. This finding points to the need for policies that ensure job security to improve staff satisfaction (Ayodele et al., 2022). Person-job fit was identified as a strong predictor of job satisfaction. Interestingly, this study also revealed gender differences in perceived job fit among administrative staff, with male staff members reporting a better fit than their female counterparts (Issah, 2021).

Leadership styles and organizational commitment were found to significantly influence job satisfaction among academic staff in Ugandan public universities, as demonstrated by Mwesigwa et al. (2020). Their study indicates that job satisfaction can mediate the relationship between leadership styles and organizational commitment.

In conclusion, the literature indicates that job satisfaction among university faculty and administrative staff is influenced by a complex interplay of factors including organizational support, HR practices, job security, organizational culture, and leadership styles. Understanding and addressing these factors is crucial for enhancing job satisfaction and, by extension, the overall effectiveness of academic institutions.

### Mediating role of organizational justice perceptions

As mentioned above, Shahid et al. (2022) explored the impact of high-performance work systems on job satisfaction in universities, emphasizing the significant role of perceived organizational support. Their findings suggest that when faculty and staff perceive their organization as supportive, it enhances job satisfaction. This perception acts as a crucial moderator in the relationship between work systems and employee satisfaction. The study highlights that the presence of a supportive and fair work environment is essential for fostering job satisfaction among university employees, suggesting that perceived organizational support can serve as a form of organizational justice.

Furthermore, foundational research provided an understanding of how organizational justice relates to stress and work–family conflict. Their research indicates that fair treatment within the university significantly reduces stress levels among faculty. This reduction in stress, mediated by a decrease in work–family conflict, leads to improved job satisfaction. This finding is particularly relevant, as it suggests that perceptions of fairness in organizational procedures and interactions can help manage the balance between work and family life, thereby positively impacting job satisfaction (Judge and Colquitt, 2004).

These studies collectively underscore the importance of organizational justice in the academic environment. They highlight that the perceptions of fairness and support within the organization not only directly influence job satisfaction but also play a vital role in mediating the effects of work-family dynamics on employee wellbeing. The implications of these findings are significant for university administrations aiming to enhance job satisfaction and overall wellbeing among their staff. By fostering a just and supportive work environment, universities can positively impact both the professional and personal lives of their employees.

### Hypothesis

Based on the above revised literature, the present study proposed the following hypotheses:

*H*1: Work-Family Culture will predict job satisfaction among Faculty (H1a) and Administrative staff (H1b).

*H*2: Organizational Justice perceptions will mediate the relationships between Work-Family Culture and Job satisfaction among Faculty (H2a) and Administrative staff (H2b).

**Figure 1:**
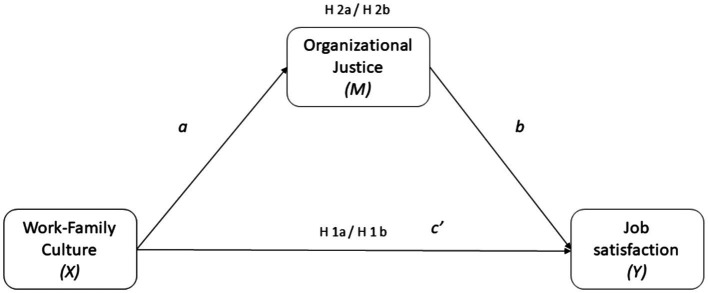
Conceptual model and hypotheses for the current study. Indirect effect of X through M = a_1_b_1._ Direct effect of X on Y = c’.

The full model of hypotheses is displayed in Figure 1.

## Methods

### Participants

This research was conducted using two distinct groups. The first group consisted of 1,075 faculty members at Chinese universities, while the second group comprised 972 administrative and technical employees at these institutions. Within the faculty group, 43.4% identified as male. Conversely, in the administrative and technical staff group, 61.9% were female. Regarding educational attainment, a significant majority of the faculty members (81.3%) held Ph.D. degrees, followed by 10.8% with Master’s degrees and 6% with Bachelor’s degrees. In contrast, among the administrative and technical staff, only 1.2% held Ph.D. degrees, while 46.7% had Bachelor’s degrees, 15.5% had received vocational education, and the remaining personnel had completed high-level secondary school education. The initial sample sizes for each group were larger (Administrative and Technical Staff, *N* = 1,038; Teaching Staff, *N* = 1,189). However, by the conclusion of the data collection period, which spanned 3 weeks from February 20 to March 18, 2023, any questionnaires that had not been completed to 100% were excluded. Consequently, the final sample comprised only those surveys that were fully completed.

### Procedure

The present study adopted a cross-sectional design. Ethical approval for the research was granted by the Huaiyin Institute of Technology Ethical Board. The study’s methodology involved disseminating a quantitative survey through various social networks to effectively reach the targeted groups. Potential respondents were approached with a message via LinkedIn, WeChat (primarily targeting administrative staff), and ResearchGate and Academia.edu (focusing on faculty members). This message outlined the study’s objectives, emphasized the voluntary nature of participation, assured confidentiality, and highlighted the option to withdraw at any time. It also extended an invitation to participate in the survey.

Respondents who agreed to participate were redirected to the Qualtrics website, where they first encountered questions pertaining to informed consent. Following this, they proceeded to respond to the survey. The survey was designed to be smartphone-friendly, allowing respondents the convenience of completing it on their devices. Additionally, it provided the flexibility to save progress and continue at a later time.

### Instruments

The same instruments have been used to assess the variables for both participants’ groups.

### Work-family culture

The Work-Family Culture Scale was used to assess this variable, using six items of the original scale developed by Thompson et al. (1999). The items assess respondents’ perceptions of the overall extent to which their organizations facilitate employees’ efforts to balance work and family responsibilities. Two items belong to the managerial support factor, two items to the second factor (negative career consequences associated with devoting time to family responsibilities, reversed items), and the last two items to the factor focused on the organizational time demands or expectations that may interfere with family responsibilities. The six items have been selected by choosing those that showed higher loadings on each factor in the original study. The items were: (Managerial support) *In general, managers in this organization are quite accommodating of family-related needs; Higher management in this organization encourages supervisors to be sensitive to employees’ family and personal concerns;* (Negative career consequences) *Many employees are resentful when men in this organization take extended leaves to care for newborn or adopted children. (R) To turn down a promotion or transfer for family-related reasons will seriously hurt one’s career progress in this organization. (R)* (Organizational time demands) *Employees are often expected to take work home at night and/or on weekends. (R);* and *To be viewed favorably by top management, employees in this organization must constantly put their jobs ahead of their families or personal lives. (R).*

### Organizational justice

One of the most widely used questionnaires to assess Organizational justice in China is the Perceived Organizational Justice Scale, that consists of 20 items (Niehoff and Moorman, 1993). In the original version, of the 20 items, 5 items measured distributive justice (e.g., “I consider my workload to be quite fair”), 6 items measured procedural justice (e.g., “Job decisions are made by the general manager in an unbiased manner.”), 9 items measured interactional justice (e.g., “When decisions are made about my job, the general manager treats me with kindness and consideration”). This scale is frequently used in China and has good validity and reliability (Ouyang et al., 2015). In the present study, nine items of the original scale were used, the three that showed highest factor loadings in each dimension. As in previous studies, the items were used to obtain a general Organizational justice perception (Shen et al., 2022).

### Job satisfaction

The Job Satisfaction Scale-Chinese Version (Xie et al., 2017), was used in the present study. It consists of 3 Likert-scaled items (e.g., “All things considered, I feel pretty good about this job”), that loaded in a single factor of general job satisfaction.

### Sociodemographic information

Participants’ gender (male, female, refuse), age and organizational seniority as well as unit seniority have been assessed, as number of years.

### Statistical analysis

Descriptive and correlational analyses were conducted with SPSS 24, and the Process macros for SPSS (version 4.2) has been used for mediational analyses proposed by Hayes (2022).

The mediating effect analysis allows to investigate how the predictor affects the criterion variable. In particular, the hypothesized relationships were assessed using Model 4, which estimates the indirect effect of X (Work-Family culture) on Y (Job satisfaction) through M (Organizational justice) both in the sample of Faculty and the Administrative Staff. This procedure was based on 5,000 bootstrap re-samples. When zero is not included in the 95% bias-corrected confidence interval, it may be concluded that the parameter is significantly different from zero at *p* < 0.05. Calculation of the Indirect effect of *X* through *M* = *a_1_b_1_*, and the direct effect of *X* on *Y* = *c’* (see Figure 1).

## Results

Among the faculty members. The data indicates significant relationships between several factors, as Table 1 shown. There is a negative correlation between Age and Work-Family Culture. Organizational Seniority. while strongly linked with Unit Seniority. does not show a strong relationship with Organizational Justice. A moderate positive correlation between Work-Family Culture and Organizational Justice has been shown. Additionally, both these factors show a positive correlation with Job Satisfaction, providing preliminary support for the study hypotheses.

**Table 1:** Descriptive statistics and Pearson Correlations Faculty members (*N* = 1,075).

Variables	Mean	SD	1	2	3	4	5	6
1. Age	3.59	1.16	1					
2. Organizational seniority	3.11	1.67	0.69^**^	1				
3. Unit seniority	2.91	1.59	0.64^**^	0.88^**^	1			
4. Work-family culture	3.99	1.09	−0.12^**^	−0.14^**^	−0.15^**^	*0.86*		
5. Organizational justice	3.60	1.21	0.15^**^	0.04	0.08^**^	0.39^**^	*0.82*	
6. Job satisfaction	5.34	1.04	0.08^**^	−0.02	−0.02	0.39^**^	0.60^**^	*0.77*

**p < 0.01. Values in italics in the diagonal for the Cronbach’s Alphas.

As Table 2 displays, among the administrative and technical staff, the data reveals significant relationships among various workplace factors. Age shows a positive correlation with both Organizational and Unit Seniority. However, the relationship between Age and Organizational Justice is only slight. Organizational Seniority shows a negative correlation with both Work-Family Culture and Job Satisfaction. Unit Seniority also negatively correlates with Work-Family Culture and Job Satisfaction. Work-Family Culture positively correlates with both Organizational Justice and Job Satisfaction. As in the faculty members’ sample, the strongest correlation in the dataset is between Organizational Justice and Job Satisfaction.

*H*1a: *Work-Family Culture will predict job satisfaction among Faculty.*

**Table 2:** Descriptive statistics and Pearson Correlations Technical Staff (*N* = 972).

Variables	Mean	SD	1	2	3	4	5	6
1. Age	3.21	0.82	1					
2. Organizational seniority	2.89	1.55	0.55^**^	1				
3. Unit seniority	2.11	1.34	0.43^**^	0.66^**^	1			
4. Work-family culture	4.20	1.21	−0.05	−0.18^**^	−0.12^**^	*0.89*		
5. Organizational Justice	3.76	1.12	0.06^*^	−0.05	0.00	0.30^**^	*0.87*	
6. Job satisfaction	5.25	0.99	−0.01	−0.18^**^	−0.12^**^	0.34^**^	0.50^**^	*0.78*

*p < 0.05; **p < 0.01. Values in italics in the diagonal for the Cronbach’s Alphas.

Among faculty members, the results indicate a significant relationship, with Work-Family Culture positively predicting Organizational Justice, as Table 3 shows. Age shows a positive effect, while Organizational Seniority exhibits a negative effect. Unit Seniority also positively influences Organizational Justice. In the model predicting Job Satisfaction, as Table 4 displays, both Work-Family Culture and Organizational Justice emerge as significant predictors. Organizational Justice shows a stronger effect compared to Work-Family Culture. Age has a smaller positive effect, while Unit Seniority negatively influences Job Satisfaction. Organizational Seniority does not show a significant effect.

*H*1b: *Work-Family Culture will predict job satisfaction among Administrative staff.*

**Table 3:** Organizational justice as a function of work-family culture, age, and organizational and unit seniority (*N* = 1,075).

Variable	*B*	SE	*β*	*t*(1,070)	*p*	95% CI
Constant	0.917	0.172		5.34	< 0.001	[0.580, 1.255]
Work-family culture	0.469	0.031	0.460	15.35	< 0.001	[0.409, 0.529]
Age	0.254	0.040		6.42	< 0.001	[0.176, 0.331]
Organizational seniority	−0.190	0.045		−4.19	< 0.001	[−0.279, −0.101]
Unit seniority	0.168	0.045		3.73	0.0002	[0.080, 0.257]

*R* = 0.460; *R*^2^ = 0.212; MSE = 1.175; *F*(4, 1,070) = 71.93, *p* < 0.001.

**Table 4:** Job satisfaction as a function of work-family culture, age, and organizational and unit seniority.

Variable	*B*	SE	*β*	*t*(1069)	*p*	95% CI
Constant	2.91	0.12		22.54	< 0.001	[2.66, 3.17]
Work-family culture	0.17	0.02	0.63	6.90	< 0.001	[0.12, 0.22]
Age	0.45	0.02		19.88	< 0.001	[0.40, 0.49]
Organizational seniority	0.08	0.03		2.76	0.006	[0.02, 0.14]
Unit seniority	0.00	0.03		0.12	0.908	[−0.06, 0.07]

*R* = 0.63; *R*^2^ = 0.40; MSE = 0.65; *F*(5, 1,069) = 144.67, *p* < 0.001,

Among the administrative and technical staff, Work-Family Culture significantly predicts Organizational Justice as Table 5 shows. Age shows a positive influence, whereas organizational tenure has a slight negative impact. Unit tenure does not significantly affect Organizational Justice. In the model predicting Job Satisfaction, displayed in Table 6, Work-Family Culture and Organizational Justice are significant predictors. Organizational Justice has a stronger influence than Work-Family Culture. Age shows a small positive effect, while organizational tenure negatively influences Job Satisfaction. Unit tenure does not have a significant impact.

*H*2a: *Organizational Justice perceptions will mediate the relationships between Work-Family Culture and Job satisfaction among Faculty.*

**Table 5:** Effects of work-family culture, age, and seniority on organizational justice (*N* = 972).

Variable	*B*	SE	*t*(967)	*p*	95% CI
Constant	2.22	0.19	11.80	< 0.001	[1.85, 2.58]
Work-family culture	0.28	0.03	9.70	< 0.001	[0.22, 0.33]
Age	0.15	0.05	2.94	0.003	[0.05, 0.25]
Organizational seniority	−0.06	0.03	−1.93	0.054	[−0.13, 0.00]
Unit seniority	0.04	0.03	1.18	0.239	[−0.03, 0.11]

*R* = 0.32; *R*^2^ = 0.10; MSE = 1.14; *F*(4, 967) = 27.76, *p* < 0.001.

**Table 6:** Effects of work-family culture, age, and seniority on job satisfaction.

Variable	*B*	SE	*t*(967)	*p*	95% CI
Constant	4.12	0.16	25.24	< 0.001	[3.80, 4.44]
Work-family culture	0.26	0.02	10.31	< 0.001	[0.21, 0.31]
Age	0.12	0.04	2.85	0.005	[0.04, 0.21]
Organizational seniority	−0.11	0.03	−3.82	< 0.001	[−0.16, −0.05]
Unit seniority	−0.01	0.03	−0.46	0.646	[−0.07, 0.05]

*R* = 0.37; *R*^2^ = 0.14; MSE = 0.86; *F*(4, 967) = 38.81, *p* < 0.001.

The analysis conducted with the Faculty sample revealed a significant total effect of Work-Family Culture on Job Satisfaction (*B* = 0.38, SE = 0.02, *t* = 14.49, *p* < 0.001, 95% CI [0.33, 0.43], completely standardized effect = 0.40), indicating a notable impact. The direct effect of Work-Family Culture on Job Satisfaction remained significant (*B* = 0.17, SE = 0.02, *t* = 6.90, *p* < 0.001, 95% CI [0.12, 0.22], completely standardized effect = 0.18), even when controlling for Organizational Justice, albeit reduced compared to the total effect. This suggests that Work-Family Culture directly influences Job Satisfaction beyond its impact through Organizational Justice.

Further, the indirect effect of Work-Family Culture on Job Satisfaction through Organizational Justice was significant (Effect = 0.21, BootSE = 0.01, 95% BootCI [0.17, 0.24]), with a completely standardized indirect effect of 0.22 (BootSE = 0.01, 95% BootCI [0.19, 0.25]). This indicates that a portion of Work-Family Culture’s effect on Job Satisfaction is mediated through its impact on perceptions of Organizational Justice, underscoring the importance of organizational perceptions in the relationship between Work-Family Culture and Job Satisfaction.

*H*2b: *Organizational Justice perceptions will mediate the relationships between Work-Family Culture and Job satisfaction among Administrative staff.*

Among the administrative staff, the total effect of Work-Family Culture on Job Satisfaction is significant (*B* = 0.25, SE = 0.02, *t* = 10.31, *p* < 0.001, 95% CI [0.20, 0.30], completely standardized effect = 0.31), showing that Work-Family Culture substantially impacts Job Satisfaction. This finding underscores the potent influence that perceptions of organizational support for balancing work and family demands have on employees’ overall job satisfaction.

There is also a significant direct effect of Work-Family Culture on Job Satisfaction (*B* = 0.14, SE = 0.02, *t* = 6.38, *p* < 0.001, 95% CI [0.10, 0.19], completely standardized effect = 0.18), indicating that Work-Family Culture influences Job Satisfaction even when accounting for Organizational Justice. This suggests that aspects of Work-Family Culture directly contribute to how satisfied employees feel at their job, independent of how justly they perceive their organization’s policies and practices.

Moreover, the analysis reveals a significant indirect effect of Work-Family Culture on Job Satisfaction through Organizational Justice (Effect = 0.10, BootSE = 0.01, 95% BootCI [0.07, 0.14]). The completely standardized indirect effect is 0.13 (BootSE = 0.01, 95% BootCI [0.09, 0.17]), highlighting the mediating role of Organizational Justice in the relationship between Work-Family Culture and Job Satisfaction. This indicates that part of the effect of Work-Family Culture on Job Satisfaction operates through its impact on employees’ perceptions of fairness and equity within the organization, reinforcing the interconnectedness between organizational practices, perceived justice, and employee satisfaction.

## Discussion

The main objective of this study was to test the mediating model of Organizational Justice into the relationship between Work-Family Culture and Job Satisfaction. Both the direct effects of Work-Family Culture and the indirect effects through Organizational Justice were significant on Job Satisfaction, providing full support for both hypotheses. Another interesting point relates to the fact that the findings were similar among the faculty and the administrative and technical staff of different academic institutions. Despite that some scholars found differences between these two occupational groups based on their job characteristics (Rosser, 2003), our research highlights that similar features, as management practices regarding the work-family interface, could be perceived for the individuals in a similar way (Chung et al., 2010). This research offers insights into the relationships between Work-Family Culture, Organizational Justice, and Job Satisfaction within academic institutions, and some findings deserve further discussion.

First, the generational differences in Work-Family Culture perception, as indicated by the negative correlation with age among faculty members, highlight a shifting paradigm in work-life balance. This suggests that younger faculty may place greater emphasis on work-family integration than their older counterparts, potentially reflecting broader societal changes (Alcover et al., 2023).

Second, the lack of a strong relationship between Organizational Seniority and Organizational Justice across both faculty and administrative staff underscores a critical insight: longevity in an organization does not necessarily equate to higher perceptions of fairness and justice. This finding challenges conventional beliefs about organizational loyalty and its impact on employees’ perceptions of justice (Tsogtsuren and Tugsuu, 2016; Konrad and Bhardwaj, 2024).

Third, the moderate positive correlation between Work-Family Culture and Organizational Justice across both groups suggests that fostering a supportive work-family environment is instrumental in enhancing perceptions of organizational fairness. This is a very relevant consideration for institutions aiming to improve overall job satisfaction and organizational climate. In a similar vein, Neupane (2023) confirmed that work to family conflict and family to work conflict negatively affect job satisfaction in the Malaysian academic community, with work-family balance partially mediating the relationship between work to family conflict and job satisfaction.

Fourth, among administrative and technical staff, the negative correlation between Organizational Seniority and both Work-Family Culture and Job Satisfaction is particularly striking. It implies that increased responsibilities or evolving workplace dynamics over time may adversely affect senior employees’ work-life balance and satisfaction. This trend, mirrored in the Unit Seniority findings, points to a potential area of concern for organizational leaders. In line with our findings, previous research showed that work-family balance positively affects job satisfaction, mediated by a family supportive organizational culture (Park et al., 2020). Panda et al. (2022) also provided empirical evidence for the relationship between Work–Family Conflict and Job Satisfaction in a collectivist society, with a focus on a public sector bank in India. They explored the moderating role of nurturing task leadership behavior in attenuating the negative effects of Work–Family Conflict on job satisfaction, offering a perspective that complements the current study’s findings on the indirect effects of Work-Family Culture through Organizational Justice on Job Satisfaction.

Finally, the mediational analyses present a deeper understanding of these relationships. The significant predictive role of Work-Family Culture on Organizational Justice in both groups, coupled with its indirect impact on Job Satisfaction through Organizational Justice, underscores the pivotal role of work-family dynamics in shaping employee perceptions and satisfaction (Ouyang et al., 2015). Related to our results are previous studies that confirmed that work to family conflict and family to work conflict negatively affect job satisfaction in the Malaysian academic community, with work-family balance partially mediating the relationship between work to family conflict and job satisfaction (Rahman et al., 2020).

The stronger effect of Organizational Justice compared to Work-Family Culture in predicting Job Satisfaction highlights the critical importance of fairness and justice perceptions in the workplace. Contrary to our view, Hassan and Hashim (2011) found no significant difference in the perception of organizational justice between national and expatriate academic staff in Malaysian institutions, except for job satisfaction where Malaysians had higher satisfaction compared to expatriates. This suggests that while organizational justice perceptions might not differ significantly across nationalities, job satisfaction levels can vary, indicating other contributing factors beyond organizational justice.

The direct and indirect effects of Work-Family Culture on Job Satisfaction, even when controlling for Organizational Justice, illustrate the complex interplay between these variables. It suggests that while Organizational Justice is a significant mediator, Work-Family Culture independently contributes to Job Satisfaction. The complexities of this relationship have been proved by previous research in different cultures. The impacts of organizational justice, organizational culture, knowledge management, and employee engagement on job satisfaction among public officers in Mongolia have been examined by Tsogtsuren and Tugsuu (2016). Their findings that all these factors had positive impacts on job satisfaction suggest a more complex interplay of elements influencing job satisfaction, beyond just work-family culture and organizational justice. In a similar way, with a sample of employees from a public sector bank in India, Panda et al. (2022) found that leadership styles was another significant factor in the dynamics of work–family conflict and job satisfaction, suggesting that leadership interventions can mitigate some of the negative impacts of work–family conflict on job satisfaction.

### Limitations of the present study

The limitations of this research are noteworthy and merit careful consideration, particularly concerning its scope and applicability beyond the Chinese higher education context. This study, by design, concentrates on universities in China, a decision that, while yielding in-depth insights into the specificities of Chinese educational institutions, inherently constrains the direct applicability of our findings to other cultural and institutional frameworks. The unique cultural, administrative, and pedagogical landscapes within which Chinese universities operate may not fully represent or align with those encountered in diverse global contexts.

Consequently, the extrapolation of our conclusions to different educational systems and cultures should be approached with caution. This inherent limitation underscores the necessity of further research aimed at exploring whether, and to what extent, our findings can be observed in other settings. Comparative studies could be particularly beneficial in this regard, offering a more nuanced understanding of the universal versus context-specific dimensions of our insights. Additionally, future research could focus on identifying mechanisms through which the identified phenomena may manifest differently across various cultural and institutional backdrops.

By acknowledging these limitations, we do not diminish the value of our findings within the Chinese context but rather delineate the boundaries of their applicability and call attention to the fertile ground for further investigation. This approach not only enhances the transparency and integrity of our study but also contributes to a more informed and nuanced dialogue within the academic community about the complexities of higher education across different cultural landscapes. In this vein, an expansion or even a comparison among the relationships within different cultures would be advisable (Agarwala et al., 2020; Doblytė and Tejero, 2021; Rahim et al., 2022). Additionally, the gender distribution within the participant groups was notably skewed, with a male majority in the faculty and a female majority in the administrative staff. This imbalance could significantly influence the study outcomes, especially in aspects related to work-family culture and organizational justice, which are often perceived differently across genders. Furthermore, the contrast in educational levels between the faculty, predominantly Ph.D. holders, and the administrative staff, with lower academic qualifications, may have introduced a bias in the survey responses. This variation in educational background could affect how participants perceive and respond to questions about work-life balance and job satisfaction.

The method of participant selection and data collection also presents limitations. Using social networks for recruitment may lead to selection bias, reaching individuals more active on these platforms. The methodological framework of this study, primarily anchored in a cross-sectional design, introduces specific limitations concerning causal inference and the delineation of temporal relationships among the variables of interest—namely, work-family culture, perceptions of organizational justice, and job satisfaction. While cross-sectional analyses provide valuable snapshots of these variables and their interrelations at a single point in time, they inherently restrict our ability to infer causality or ascertain the directionality of the observed relationships. This limitation is significant, given that understanding the causal pathways between work-family culture, organizational justice perceptions, and job satisfaction is crucial for both theoretical advancement and the practical application of findings in organizational settings.

Although mediational analysis offers a methodological approach to explore potential mechanisms underlying these relationships, the cross-sectional nature of the data precludes definitive conclusions about causality. Such analyses, while insightful, cannot substitute for the temporal sequencing of variables required to establish causal links conclusively.

To overcome these limitations and build on the foundation laid by this study, future research directions should include longitudinal or experimental designs. Longitudinal studies, by tracking changes over time, would allow for a more precise examination of how the relationships between work-family culture, organizational justice perceptions, and job satisfaction evolve, thereby offering stronger evidence of causality and directionality. Experimental designs, on the other hand, could manipulate specific variables to directly observe their effects on job satisfaction and perceptions of organizational justice, further clarifying these relationships’ causal nature.

Additionally, incorporating these more robust research designs would not only validate and potentially replicate the findings of the current study but also enrich our understanding of the dynamics at play. It would allow for a nuanced exploration of the temporal precedence and causal pathways among the study variables, thereby contributing to a deeper and more accurate understanding of the interplay between organizational culture, justice perceptions, and employee satisfaction.

By acknowledging these methodological constraints and outlining future research pathways, this study underscores the importance of continued inquiry into the complex mechanisms that underpin organizational behavior. The adoption of longitudinal and experimental approaches in subsequent research efforts will be instrumental in advancing our theoretical and practical knowledge in this domain.

This study’s methodology, while robust in many respects, does encounter limitations tied to its reliance on self-reported data for assessing work-family culture, perceptions of organizational justice, and job satisfaction. Such self-report measures, albeit common and validated within social science research for their practicality and direct insight into participants’ perspectives, are susceptible to inherent biases that must be acknowledged.

Firstly, the partial use of validated survey instruments, due to the selection of specific items from the original scales, could potentially narrow the depth and breadth of the constructs being measured. This approach, while necessary to maintain the survey’s focus and manage its length, may limit the comprehensiveness of our assessment, possibly overlooking nuanced aspects of the constructs that were not covered by the selected items.

Moreover, the nature of self-report data introduces the possibility 18–20. of response biases, notably the inclination toward socially desirable responding. This bias can lead participants to overreport behaviors or attitudes perceived as favorable or underreport those deemed unfavorable, thereby skewing the findings. Such distortions are critical considerations in interpreting the study’s results, as they may reflect, to some extent, an idealized or moderated version of the participants’ true experiences or perceptions.

To mitigate these limitations and enhance the reliability of our findings, future research could incorporate alternative or supplementary measures beyond self-reports. Triangulation with qualitative data, observational methods, or objective performance indicators, for example, could provide a more multifaceted view of the constructs under study and help validate the self-reported data. Additionally, employing techniques such as the use of reverse-worded items or including measures of social desirability bias could further strengthen the study’s methodological approach by minimizing the impact of response biases.

In acknowledging these limitations, we recognize the necessity for cautious interpretation of the study’s findings. The potential biases associated with self-reported measures underscore the importance of seeking validation through alternative methodologies and underscore the need for ongoing research to corroborate and expand upon the insights gained in this study. Lastly, the study’s demographic assessment focused on basic variables such as gender, age, and seniority. However, it overlooked other potentially influential factors like marital status, parenthood, or specific job roles, which could have a substantial impact on individuals’ perceptions of work-life balance and job satisfaction.

To further the understanding of the causal relationships and directionality among the variables of interest in this study, future research should consider the incorporation of longitudinal or experimental designs. Longitudinal research allows for the tracking of changes over time, providing invaluable insights into the evolution of work-family culture, organizational justice perceptions, and job satisfaction among employees. This approach can shed light on the temporal sequence of events and the causal relationships that might exist between these variables. On the other hand, experimental designs, through the manipulation of variables within controlled environments, offer a direct way to examine causal effects, thereby enhancing our comprehension of these dynamics.

Additionally, the application of advanced statistical techniques such as structural equation modeling (SEM) is highly recommended for subsequent studies. SEM stands out for its ability to simultaneously test complex relationships between observed and latent variables, including both direct and indirect effects. This capability makes it particularly suited for exploring the intricate relationships and underlying mechanisms that govern the interactions among the study’s key constructs. Employing SEM not only aids in affirming the validity of the theoretical model proposed but also in fine-tuning our understanding of how organizational policies and culture impact employee outcomes. Through these methodological enhancements, future research has the potential to significantly enrich theoretical models and offer practical insights for improving organizational practices, thereby advancing both academic knowledge and organizational effectiveness.

### Implications for human resource managers at universities

The findings on the relationship between Work-Family Culture, Organizational Justice, and Job Satisfaction have several implications for Human Resource managers at universities. The significance of both direct and indirect effects of Work-Family Culture on Job Satisfaction through Organizational Justice provides an argument for HR managers to prioritize the development and implementation of policies that foster a supportive work-family environment. Firstly, the emphasis on creating a culture that supports work-family integration is crucial, especially in light of the generational shift toward valuing this balance. HR managers should consider initiatives such as flexible working arrangements, child care services, and family leave policies. These measures cater to the changing demographics of faculty and staff and align with broader societal changes toward work-life balance.

Secondly, the findings suggest that organizational justice is a critical factor for job satisfaction, independent of organizational seniority. This challenges the conventional belief that longer tenure and loyalty automatically lead to higher perceptions of fairness. HR managers should ensure that policies and practices promoting fairness and justice are transparent, consistently applied, and communicated effectively across all levels of the organization.

The moderate positive correlation between Work-Family Culture and Organizational Justice across different groups indicates that a supportive work-family environment enhances perceptions of organizational fairness. This insight is particularly relevant for institutions aiming to improve overall job satisfaction and organizational climate. HR managers can leverage this by implementing family-supportive policies and practices that are perceived as fair and equitable, thereby potentially improving job satisfaction.

Among administrative and technical staff, the negative correlation between Organizational Seniority and both Work-Family Culture and Job Satisfaction points to the importance of addressing the evolving workplace dynamics that may adversely affect senior employees’ work-life balance and satisfaction. HR managers should consider targeted interventions that address the unique challenges faced by senior staff, such as mentorship programs, career development opportunities, and workload management.

Lastly, the study’s mediational analyses reveal the complex relationships between Work-Family Culture, Organizational Justice, and Job Satisfaction. HR managers should recognize the relevant role of work-family dynamics in shaping employee perceptions and satisfaction. By prioritizing organizational justice and fostering a supportive work-family culture, HR managers at universities can make significant strides in enhancing job satisfaction among faculty and staff, irrespective of their generational cohort or organizational seniority. This approach not only contributes to a positive organizational climate but also supports the recruitment and retention of high-quality staff, ultimately benefiting the academic institution as a whole.

## Conclusion

This study reinforces the importance of nurturing a supportive work-family culture and promoting perceptions of organizational justice to enhance job satisfaction in academic settings. It underscores the need for policies and practices that address the evolving needs and perceptions of diverse employee groups, particularly considering generational differences and the varying impacts of organizational tenure. Such initiatives are not only crucial for employee wellbeing but are also instrumental in cultivating a positive and productive organizational climate.

## Data availability statement

The raw data supporting the conclusions of this article will be made available by the authors, without undue reservation.

## Ethics statement

The studies involving humans were approved by the Huaiyin Institute of Technology Ethical Board. The studies were conducted in accordance with the local legislation and institutional requirements. The participants provided their written informed consent to participate in this study.

## Author contributions

TH: Writing – review & editing, Writing – original draft.
